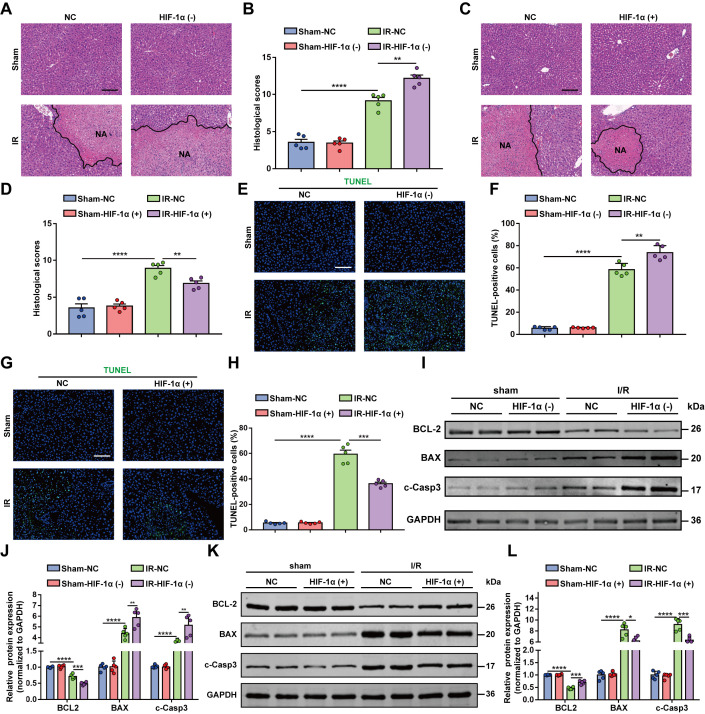# Von Hippel-Lindau deficiency protects the liver against ischemia/reperfusion injury through the regulation of hypoxia-inducible factor 1α and 2α

**DOI:** 10.1097/HC9.0000000000000567

**Published:** 2024-11-25

**Authors:** Zihao Li, Bing Yin, Yanan Xu, Chaoqun Wang, Xinglong Li, Shounan Lu, Shanjia Ke, Baolin Qian, Hongjun Yu, Miaoyu Bai, Zhongyu Li, Yongzhi Zhou, Hongchi Jiang, Yong Ma

**Affiliations:** 1 Department of Minimally Invasive Hepatic Surgery, The First Affiliated Hospital of Harbin Medical University, Harbin, China; 2 Key Laboratory of Hepatosplenic Surgery, Ministry of Education, The First Affiliated Hospital of Harbin Medical University, Harbin, China; 3 Department of Hepatopancreatobiliary Surgery, Affiliated Hangzhou First People’s Hospital, Zhejiang University School of Medicine, Hangzhou, China; 4 Department of Hepatobiliary Surgery, The Second Affiliated Hospital of Army Medical University, Chongqing, China; 5 Department of Hepatobiliary Surgery, Peking University People’s Hospital, Beijing, China

**Keywords:** hepatic ischemia/reperfusion injury, HIF-1α, HIF-2α, VHL

## Abstract

**Background:**

Ischemia and reperfusion (I/R)-induced liver injury contributes to morbidity and mortality during hepatic surgery or liver transplantation. As a pivotal regulator of cancer and inflammation, the role of Von Hippel-Lindau (VHL) in hepatic I/R injury remains undetermined.

**Methods:**

We investigated the role of VHL in hepatic I/R injury by generating VHL conditional knockout (VHL-KO) mice. The downstream mechanisms of VHL were confirmed, and the role of HIF-2α in hepatic I/R injury was further investigated.

**Results:**

In this study, we discovered that VHL upregulation was associated with hepatic I/R injury in a mouse model. VHL gene knockout (VHL-KO) and overexpression (Ad-VHL) mice demonstrated that VHL aggravated liver injury, increased inflammation, and accelerated cell death in hepatic I/R injury. The VHL protein (pVHL) regulates a crucial control mechanism by targeting HIFα subunits for ubiquitin-mediated degradation. In vitro and in vivo studies demonstrated that VHL interacted with and repressed hypoxia-inducible factor 1α (HIF-1α) and hypoxia-inducible factor 2α (HIF-2α) expression during hepatic I/R injury. Notably, the inhibition of HIF-1α or 2α, as well as the concurrent inhibition of HIF-1α and 2α, abrogated the protective effect of VHL-KO. The severe stabilization of HIF-1α or 2α, as well as the simultaneous overexpression of HIF-1α and 2α, compensated for the detrimental effect of VHL.

**Conclusions:**

Thus, we identified the VHL-HIF-1α/HIF-2α axis as an indispensable pathway that may be a novel target for mediating hepatic I/R injury.

## INTRODUCTION

Hepatic ischemia and reperfusion (I/R) injury, an innate immunity-mediated inflammatory response, is a major complication of liver transplantation, hemorrhagic shock, and partial hepatectomy.^1^ The characteristic of hepatic I/R injury is the initial limitation of blood supply to the liver (ischemia), followed by the successful reconstruction of perfusion and accompanying reoxygenation (reperfusion).^2^^,^^3^ During the ischemic period, the liver is deprived of oxygen and nutrients, leading to disturbances in cellular metabolism. Upon blood flow reperfusion, more fearful damage is induced because of the diffusion of inflammation and cell death.^4^ The important markers of liver I/R injury are sterile inflammation and cell apoptosis, which are closely related to the prognosis of liver surgery. The mechanism of liver I/R injury is extremely complicated and involves the interaction of various cell types and molecular pathways, which may explain the difficulty of developing therapeutic methods that can be translated into the clinic. Therefore, further elucidation of the molecular mechanism of hepatic I/R injury is urgently needed to identify potential targets for the development of novel therapeutic strategies.

Von Hippel-Lindau (VHL) syndrome is a cancer syndrome that is inherited predominantly in a familial manner and easily causes multiple malignant and benign tumors.^5^ VHL disease is caused by a germline mutation of the tumor suppressor gene *VHL*, which has a series of pathological features characterized by disordered cell growth, including kidney cancer, pheochromocytomas, and hemangioblastomas. Most studies have reported that the VHL protein (pVHL, the product of the *VHL* tumor suppressor gene) is the substrate recognition component for an E3-ubiquitin ligase complex, which contains elongin C, elongin B, cullin2, and the RING-box protein RBX1.^6^ The α domain of pVHL binds elongin C to form a ubiquitin complex by recruiting additional proteins, and its β domain binds the HIFα subunits in a VHL-dependent manner for ubiquitination and proteasomal destruction.^7^ pVHL connects with many other proteins consisting of HIFα and has numerous functions, including microtubule dynamics, cell proliferation, neuronal apoptosis, extracellular matrix deposition, DNA damage response, and primary cilia maintenance.^8^^,^^9^ Inactivation of pVHL results in the accumulation of HIFa subunits by mutation, and retransfer of VHL into VHL-deficient cells restrains the capacity of the cells to form tumors.^10^ VHL can even act as a key regulatory factor that participates in the development of perivascular and bronchial inflammation caused by papain challenge.^11^ Therefore, we suspect that VHL may be involved in the development of hepatic I/R injury. Nevertheless, the specific mechanism has not been investigated.

Hypoxic microenvironments are usually discovered in solid tumors, transplantation, and surgical operations and are associated with treatment failure and poor prognosis. More than 100 genes are regulated by hypoxia-inducible factor (HIF), including those involved in adjusting tumor angiogenesis, energy metabolism, and cell proliferation.^12^ HIFs are a family of transcriptional regulators characterized by high evolutionary conservation, and their function is to have a homeostatic response to low oxygen tension. Both hypoxia-inducible factor 1α (HIF-1α) and hypoxia-inducible factor 2α (HIF-2α) are stabilized and activated by hypoxia and dimerize with HIF1β. Similarly, both subtypes activate target gene transcription by binding to the same hypoxia-response element.^13^^,^^14^ However, HIF-1α and 2α are not functionally superfluous. Many studies have indicated that HIF-1α induces apoptotic pathways that are not targeted by HIF-2α and preferentially drives the expression of glycolytic pathway genes, whereas HIF-2α preferentially accelerates growth and angiogenesis.^15^^–^^17^ HIF-1α is expressed in all tissues, whereas HIF-2α is strongly expressed in the liver and intestine.^18^ HIF-1α activation has been recorded in numerous pathological conditions through different mechanisms,^19^^–^^22^ one of which is the pharmacological stabilization of HIF-1α to protect against hepatic I/R injury.^23^ Interestingly, our previous study demonstrated that HIF-1α is stabilized by VHL in hepatoma cells.^24^ However, the precise role of HIF-2α and whether HIF-1α and/or 2α require VHL to function in hepatic I/R injury remain unknown.

In this study, we revealed an intimate connection between VHL upregulation and hepatic I/R injury and revealed that HIF-2α could play a protective role. Inactivation of VHL protected against hepatic I/R injury through stabilizing HIF-1α and 2α. Therefore, we confirmed VHL as a novel protective regulatory factor in hepatic I/R injury.

## METHODS

### Human liver tissues

The human HIRI liver tissues and serum used in this study were obtained from patients who underwent partial hepatectomy for benign diseases at the First Affiliated Hospital of Harbin Medical University. No samples from executed prisoners or institutionalized persons were used in the experiments. All patients and their family members provided informed consent by signing the appropriate documentation. All the clinical samples were obtained in accordance with the ethical approval granted by the Research Ethics Committee of the First Affiliated Hospital of Harbin Medical University.

### Animals

All the mice (male, 8–10 wk old, 25 ± 2 g) were housed in a specific pathogen-free and suitable temperature environment under a 12-hour light/dark cycle, and they had access to food and water freely throughout the study period. All surgical procedures and care administered to the animals were approved by the Institutional Animal Care and Use Committee of Harbin Medical University.

*VHL*-floxed mice (Flox) were generated through Cre/lox site–specific recombination technology. To generate *VHL* conditional knockout (*VHL-*KO) mice in which *VHL* is inactivated in multiple tissues in an inducible manner, we crossed *VHL*^*f/f*^ mice with *VHL*^*d/+*^ mice carrying the tamoxifen-inducible Cre recombinase transgene (*VHL*^*d/+*^/*CreER*^*TM*^) on the C57BL/6 background. Both types of mice were purchased from Cyagen Biosciences Company. For our experiments, *VHL*^*f/d*^/*CreER*^*TM*^ mice were injected i.p. with tamoxifen in corn oil (0.36 mg/g body weight) to activate Cre recombinase 1 week before hepatic I/R injury. Male *VHL*^*f/d*^ */CreERTM* mice and littermate *VHL*^*f/+*^ */CreERTM* control mice were subjected to tamoxifen induction for these experiments.

### Adenoviral vector construction and transduction

The expression plasmids pcDNA3 HIF-1α TM (a mutant with the triple mutation [TM] P402A/P577A/N813A to make the mouse HIF-1α protein normoxia-active) and pcDNA3 HIF-2α TM (a mutant with the triple mutation [TM] P405A/P530A/N851A to make the mouse HIF-2α protein normoxia-active) were used in this experiment.^24^ Adenoviral vectors delivering HIF-1α TM (Ad-HIF-1α TM) and HIF-2α TM (Ad-HIF-2α TM) were purchased from GeneChem. An Ad-Null vector was used as a negative control. Two adenoviral vectors delivering siRNAs that target mouse HIF-1α (AV-siHIF-1α) and HIF-2α (AV-siHIF-2α) were purchased from GeneChem. An adenoviral vector expressing a scrambled RNA was used as the control.

To achieve intrahepatic VHL overexpression and HIF-1α and HIF-2α overexpression and knockdown, Ad-VHL, HIF-1α (+), HIF-1α (−), HIF-2α (+), and HIF-2α (−) were delivered to mice through tail vein injection with 1×10^9^ TCID50/mouse. Ad-Null and the indicated NC were used as negative controls. After 72 hours, the liver I/R procedures were carried out. For the in vitro experiments, primary hepatocytes were cultured with HIF-1α (+) and HIF-2α (+), HIF-1α (−) and HIF-2α (−), and the indicated NC for 2 hours at an MOI of 100, and the functional assays were carried out 48 hours later. The primers of genotyping and siRNA target sequences are listed in Supplemental Table S1 http://links.lww.com/HC9/B75.

### Mouse liver I/R injury model

We used a partial liver warm hepatic I/R injury model as described.^25^ In general, the mice were anesthetized with pentobarbital sodium (50 mg/kg). A midline laparotomy was subsequently performed to expose the liver, and an atraumatic microvascular clip clamped the portal vein, hepatic artery, and bile duct to interrupt the blood supply to the left lateral/median lobes (~70%) of the liver. After ischemia for 90 minutes, the clamp was released for reperfusion. At different times of reperfusion, the mice were anesthetized, and liver samples and serum were collected for further analysis. Sham control mice underwent the same operative procedure without vasculature occlusion.

### Cell culture and hepatocyte hypoxia/reoxygenation model

Primary hepatocytes were isolated as described.^26^ The isolated cells were seeded and cultured overnight in complete DMEM/F12. After the medium was changed to serum-free and glucose-free DMEM/F12, hypoxic conditions were created in a modular incubator and maintained by continuous gas flow with a 1% O_2_, 5% CO_2_, and 94% N_2_ gas mixture. After 60 minutes of hypoxia, the cells were incubated under normal conditions (95% air and 5% CO_2_) for the indicated times.

The specific inhibitor PT-2399 used to suppress HIF-2α was added to the culture medium of *VHL*-KO or the corresponding control hepatocytes before hypoxia/reoxygenation (H/R). The same volume of DMSO was used as a vehicle control.

### Liver biochemical measurement and determination of inflammatory factors

The extent of liver damage in the animals was evaluated by measuring the activities of ALT and AST in the serum with the ADVIA 2400 Chemistry System (Siemens) according to the manufacturer’s protocols.

The serum and medium concentrations of cytokines and chemokines were measured through commercial ELISA kits (Peprotech; R&D Systems) according to the manufacturer’s protocol.

### Histology and immunofluorescence staining

Liver samples were fixed with formalin. After deparaffinization and rehydration, the paraffin-embedded liver sections (4 μm) were stained with hematoxylin and eosin to visualize the pattern in the necrotic areas of the liver. Images were observed and captured through a light microscope (Olympus).

The TUNEL (TdT-mediated dUTP nick-end labeling) method (Roche, 11684817910) was used to detect apoptosis in paraffin-embedded liver sections and to evaluate liver injury according to the manufacturer’s protocol. Nuclei were labeled with DAPI. Images were acquired under a fluorescence microscope (OLYMPUS BX51TRF) with DP2-BSW software (version 2.2). The images were analyzed with Image-Pro Plus (version 6.0).

### Cell counting kit-8 and lactate dehydrogenase assays

Cell viability was determined with a cell counting kit-8 (CK04; Dojindo) according to the manufacturer’s instructions. A SpectraMax i3x (Molecular Devices) at 450 nm was used to assess cell viability.

A Cytotoxicity LDH Assay Kit-WST (CK12; Dojindo) was used to measure the lactate dehydrogenase (LDH) released into the media from damaged cells according to the manufacturer’s protocol.

### Quantitative real-time PCR

Quantitative real-time PCR was performed as described.^27^ Total RNA was extracted from liver tissues or cultured cells through the AxyPrep Multisource Total RNA Miniprep Kit (30318KD1, Corning). cDNA was synthesized with RNA (2 μg) through the Transcriptor First Strand cDNA Synthesis Kit (4896866001; Roche) according to the manufacturer’s instructions. Quantitative real-time PCR was performed with SYBR Green PCR Master Mix (04887352001; Roche) according to the manufacturer’s instructions. The mRNA expression was normalized to the expression of glyceraldehyde-3-phosphate dehydrogenase. The primer sequences of the target genes are listed in Supplemental Table S2, http://links.lww.com/HC9/B75.

### Western blot analysis

Protein levels were detected by Western blot analysis as described.^28^ Liver tissues or cells were homogenized in protein lysis buffer, and debris was removed by centrifugation. The protein concentration was measured through a BCA protein assay kit (080719191113; Beyotime). The protein mixture was mixed with SDS loading buffer and boiled at 95°C for 10 minutes. Proteins separated by SDS-polyacrylamide gel electrophoresis were transferred to NC membranes and blocked with 5% milk. Then, the cells were incubated with primary and secondary antibodies. DAPDH was used as an internal control. The antibodies used in this study are listed in Supplemental Table S3, http://links.lww.com/HC9/B75.

### Statistical analysis

The protein levels were detected by Western blot analysis, as previously reported. All values in this study are expressed as the means ± SDs and were analyzed using SPSS 17.0 software. Statistical differences between more than 2 groups were determined by one-way ANOVA followed by a Bonferroni post hoc correction (assuming equal variances) or Tamhane’s T2 test. The statistically significant difference between the 2 individual groups was determined with a 2-tailed Student *t* test. Differences with *p* < 0.05 were considered significant.

## RESULTS

### VHL upregulation correlates with hepatic I/R injury

**FIGURE 1:**
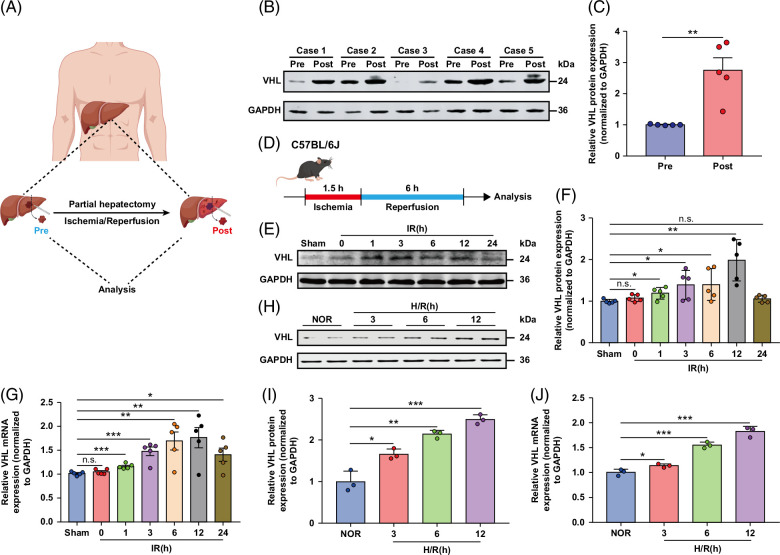
VHL is upregulated in hepatic I/R injury. (A) Liver tissues were obtained from patients who underwent partial hepatectomy for benign diseases. (B) Western blot showing VHL protein expression in the livers of benign tumor partial hepatectomy patient samples and control samples. (C) Quantification of the VHL gray value relative to that of GAPDH. (D) Experimental strategy in a mouse model of hepatic I/R injury. The mice underwent LAD coronary artery occlusion for 60 minutes, followed by reperfusion for the indicated times. (E) The levels of VHL protein in the livers of mice subjected to control conditions or 90 minutes of ischemia followed by reperfusion for the indicated periods. (F) Quantification of the VHL gray value relative to that of GAPDH. (G) *VHL* mRNA levels in the livers of mice subjected to control conditions or 90 minutes of ischemia followed by reperfusion for the indicated times. (H) Western blot showing VHL protein expression in primary hepatocytes subjected to sham or H/R treatment for the indicated times. (I) Quantification of the VHL gray value relative to that of GAPDH. (J) RT-PCR showing *VHL* mRNA levels in primary hepatocytes subjected to sham or H/R treatment for the indicated times (representative of 3 independent experiments). GAPDH served as the loading control (n = 5/group). All the data are presented as the means ± SDs. The levels of statistical significance are indicated as follows: *p < 0.05, **p < 0.01, ***p < 0.001 and ns = not significant. For statistical analysis, 1-way ANOVA with Bonferroni post hoc correction or Tamhane’s T2 post hoc test and 2-tailed Student *t* test were used. Abbreviations: GAPDH, glyceraldehyde-3-phosphate dehydrogenase; H/R, hypoxia/reoxygenation; I/R, ischemia and reperfusion; LAD, left anterior descending; RT-PCR, real-time polymerase chain reaction; VHL, Von Hippel-Lindau.

To analyze whether VHL is associated with hepatic I/R injury, we first assessed VHL expression in the livers of patients who underwent benign tumor partial hepatectomy (Figure 1A). Notably, obvious upregulation of the VHL protein was observed in the partial hepatectomy patient samples compared with the control samples (Figures 1B, C). We further explored VHL expression in wild-type (WT) mice subjected to partial hepatic I/R injury (Figure 1D). The protein and mRNA levels of VHL in the liver lobes of WT mice were distinctly increased from 1 to 24 hours of hepatic I/R treatment (Figures 1E–G). In accordance with the in vivo results, visibly increased VHL protein and mRNA levels were also observed in primary hepatocytes subjected to 1–12 hours of H/R treatment (Figures 1H–J). In conclusion, these results suggest that VHL upregulation in hepatocytes is associated with hepatic I/R injury and that VHL plays an important role in this process.

### VHL deficiency alleviates liver damage and the inflammatory response during hepatic I/R injury

**FIGURE 2:**
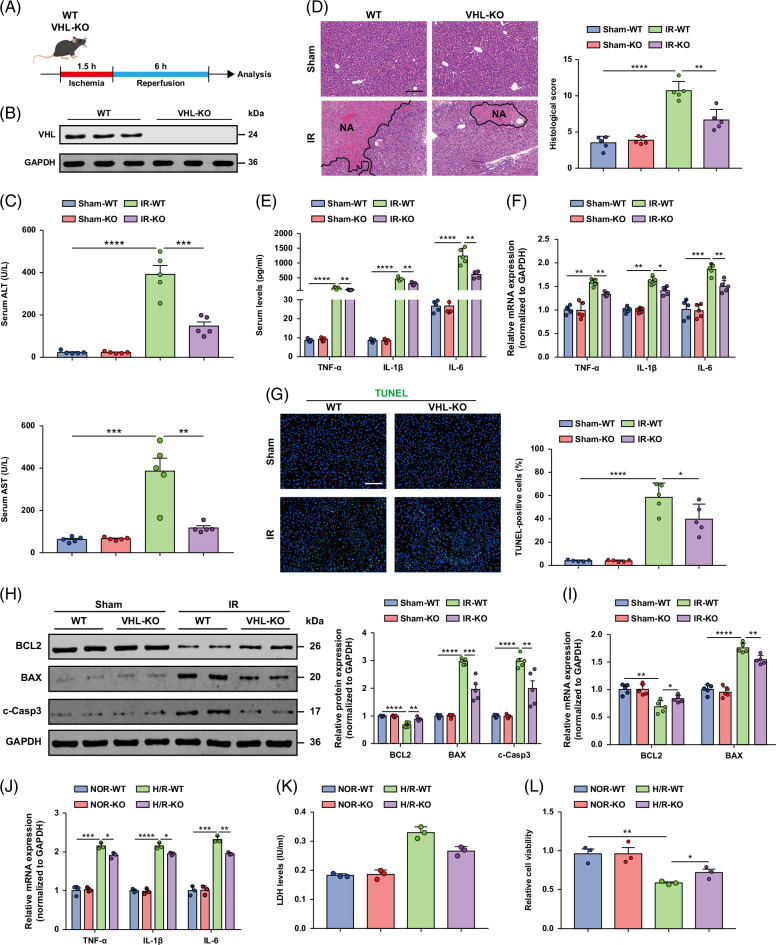
VHL deletion alleviates liver damage, the inflammatory response, and apoptosis during hepatic I/R injury. (A) Experimental strategy for generating a mouse model of hepatic I/R injury in VHL-KO and WT mice. (B) The protein expression level of VHL in the livers of *VHL*-KO mice and the corresponding control mice. (C) Serum ALT/AST activities in WT and *VHL*-KO mice at 6 hours after hepatic I/R surgery. (D) Representative histological H&E-stained images showing necrotic areas in liver tissues from WT and *VHL*-KO mice subjected to the sham or I/R operation. Scale bar, 100 μm. (E) The levels of inflammatory cytokines/chemokines in the serum of *VHL*-KO and WT mice after I/R insult. (F) The mRNA expression levels of inflammatory cytokine/chemokine genes in the livers from the indicated groups. (G) TUNEL staining of liver sections from WT and *VHL*-KO mice after the I/R operation. Scale bar, 50 μm. (H, I) The protein (H) and mRNA (I) levels of cell apoptosis–related genes in the livers of WT and *VHL*-KO mice after I/R surgery (n = 5/group). (J) The mRNA levels of inflammatory cytokines/chemokines in sham and *VHL*-KO primary hepatocytes challenged with H/R injury. (K) LDH release assay of hepatocytes from *VHL*-KO and sham hepatocytes after H/R injury. (L) The cell viability indices of *VHL*-KO and sham hepatocytes after H/R injury were determined through a CCK-8 assay. Data are representative of 3 independent experiments. GAPDH was used for normalization. All the data are presented as the means ± SDs. The levels of statistical significance are indicated as follows: *p < 0.05, **p < 0.01, ***p < 0.001, ****p < 0.0001 and ns = not significant. For statistical analysis, 1-way ANOVA with Bonferroni post hoc correction or Tamhane’s T2 post hoc test and 2-tailed Student *t* test were used. Abbreviations: CCK-8, cell counting kit-8; GAPDH, glyceraldehyde-3-phosphate dehydrogenase; H&E, hematoxylin and eosin; H/R, hypoxia/reoxygenation; I/R, ischemia and reperfusion; LDH, lactate dehydrogenase; TUNEL, TdT-mediated dUTP nick-end labeling; VHL, Von Hippel-Lindau; VHL-KO, VHL gene knockout; WT, wild-type.

As the expression level of VHL was significantly increased during liver I/R injury, we hypothesized a functional correlation between VHL and liver I/R injury (Figure 2A). To compare VHL protein expression in tamoxifen-treated VHLf/d/CreERTM (VHL-KO) mice and tamoxifen-treated VHLf/+/CreERTM (control) mice, the levels of VHL protein in murine livers after tamoxifen induction were confirmed by Western blot analysis (Figure 2B). Compared with the WT control, VHL deficiency significantly decreased the serum levels of ALT and AST at 6 hours after liver I/R surgery (Figure 2C). Hematoxylin and eosin staining revealed a considerably reduced necrotic area in VHL-KO mice subjected to hepatic I/R injury compared with WT controls (Figure 2D). These findings show that VHL deficiency alleviates I/R-mediated liver insult. During hepatic I/R injury, the inflammatory response starts at the ischemic stage and persists throughout the whole process, which is an important factor of liver I/R injury. Therefore, we wanted to investigate whether VHL affects the inflammatory response after hepatic I/R injury in mice. Compared with serum from control mice, serum from VHL-KO mice contained lower levels of inflammatory cytokines/chemokines, including TNF-α, IL-1β, and IL-6 (Figure 2E). The mRNA levels of inflammatory cytokines/chemokines in the liver tissues were significantly lower in the VHL-KO group than in the control group (Figure 2F). Compared with control primary hepatocytes, VHL-KO primary hepatocytes presented lower mRNA levels of inflammatory factors after the H/R challenge (Figure 2J). Therefore, these findings indicate that VHL regulates the inflammatory response and that VHL knockout reduces liver injury during hepatic I/R injury.

### VHL deficiency weakens apoptosis during hepatic I/R injury

Excessive inflammation induced by hepatic I/R leads to liver cell death.^29^ Thus, we examined whether VHL directly regulates apoptosis during this process. TUNEL staining revealed that the number of apoptotic cells in the livers of VHL-KO mice was significantly lower than that in the livers of WT mice after liver I/R injury (Figure 2G). Compared with those in WT mice, the expression of the antiapoptotic gene Bcl-2 (B-cell lymphoma 2) was significantly increased, and the levels of the proapoptotic genes Bax (Bcl-2-associated x protein) and c-caspase-3 were reduced in the livers of VHL-KO mice during I/R injury (Figure 2H). Moreover, the expression trends of the apoptosis-related mRNAs Bcl-2 and Bax were similar to those of the protein levels in the livers of VHL-KO mice compared with those in the livers of WT mice during I/R injury (Figure 2I). We also detected less LDH release from VHL-KO hepatocyte cultures than from control cultures after H/R injury (Figure 2K). Furthermore, inhibition of VHL increased the viability of primary hepatocytes challenged by H/R insult (Figure 2L). These observations suggest that VHL deficiency suppressed apoptosis during liver I/R injury.

### VHL overexpression exacerbates liver damage and inflammation induced by hepatic I/R insult

**FIGURE 3:**
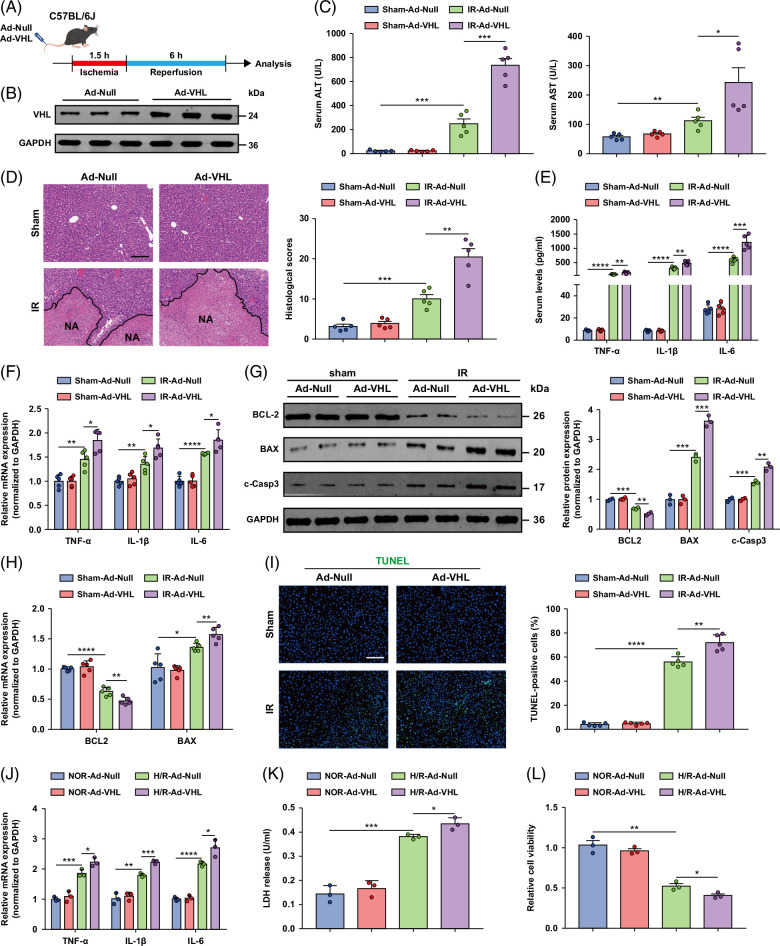
Overexpression of VHL exacerbates liver damage, inflammation, and apoptosis induced by hepatic I/R insult. (A) Experimental strategy in a mouse model of hepatic I/R injury with Ad-VHL and Ad-Null mice. (B) The protein expression level of VHL in the livers of Ad-VHL mice and the corresponding control mice. (C) Serum ALT/AST activities in Ad-Null and Ad-VHL mice after hepatic I/R surgery. (D) Representative histological H&E-stained images showing necrotic areas in liver tissue from Ad-Null and Ad-VHL mice after hepatic I/R surgery. Scale bar, 100 μm. (E) The levels of inflammatory cytokines/chemokines in the serum of Ad-Null and Ad-VHL mice after I/R insult. (F) The mRNA expression levels of inflammatory cytokine/chemokine genes in the livers from the indicated groups. (G, H) The protein (G) and mRNA (H) levels of cell apoptosis–related genes in the livers of Ad-Null and Ad-VHL mice after I/R surgery (n = 5/group). (I) TUNEL staining of liver sections from Ad-Null and Ad-VHL mice after the I/R operation. Scale bar, 50 μm. (J) The mRNA levels of inflammatory cytokine/chemokine genes in sham and VHL-overexpressing hepatocytes challenged with H/R injury. (K) LDH release assay of hepatocytes from VHL-overexpressing and sham hepatocytes after H/R injury. (L) The cell viability indices of VHL-overexpressing and sham-treated hepatocytes after H/R injury were determined through a CCK-8 assay. Data are representative of 3 independent experiments. GAPDH was used for normalization. All the data are presented as the means ± SDs. The levels of statistical significance are indicated as follows: *p < 0.05, **p < 0.01, ***p < 0.001, ****p < 0.0001 and ns = not significant. For statistical analysis, 1-way ANOVA with Bonferroni post hoc correction or Tamhane’s T2 post hoc test and 2-tailed Student *t* test were used. Abbreviations: CCK-8, cell counting kit-8; GAPDH, glyceraldehyde-3-phosphate dehydrogenase; H&E, hematoxylin and eosin; H/R, hypoxia/reoxygenation; I/R, ischemia and reperfusion; LDH, lactate dehydrogenase; TUNEL, TdT-mediated dUTP nick-end labeling; VHL, Von Hippel-Lindau.

Since VHL deficiency protects the liver from I/R-induced insult, we tested whether VHL overexpression exacerbates liver I/R injury (Figure 3A). We constructed hepatic VHL-overexpressing (Ad-VHL) mice by injecting adenovirus into the tail vein, and Western blot analysis confirmed that VHL was overexpressed in the livers of Ad-VHL mice. An Ad-Null vector was used as a negative control (Figure 3B). As expected, the ALT and AST activities of Ad-VHL mice were greater than those of Ad-Null mice after liver I/R surgery (Figure 3C). Histological analysis of the liver also revealed that Ad-VHL mice presented greater necrotic areas after liver I/R surgery than Ad-Null mice did (Figure 3D). Compared with Ad-Null mice, Ad-VHL mice presented increased levels of proinflammatory cytokines/chemokines during hepatic I/R surgery (Figures 3E, F). To further investigate whether VHL affects inflammation in hepatocytes, we used VHL-overexpressing primary hepatocytes through adenovirus infection of mouse tail veins. Overexpression of VHL aggravated the H/R-triggered inflammatory response in hepatocytes (Figure 3J). Together, these results suggest that VHL overexpression exacerbates liver damage and inflammation induced by hepatic I/R treatment.

### Overexpression of VHL aggravates apoptosis in hepatic I/R injury

It has been determined that VHL deficiency alleviates cell apoptosis induced by liver I/R injury, and we next explored the effect of VHL overexpression on cell apoptosis after liver I/R injury. TUNEL staining revealed that compared with Ad-Null control mice, Ad-VHL model mice exhibited severe cell apoptosis after liver I/R insult and similar tendencies toward increased levels of the proapoptotic molecules Bax and c-caspase-3 and decreased levels of the antiapoptotic molecule Bcl-2 (Figures 3G–I). Consistent with the results of the in vivo studies, hepatocyte apoptosis was also aggravated in VHL-overexpressing hepatocytes induced by H/R compared with control hepatocytes (Figures 3K, L). Collectively, these data strongly demonstrate that VHL regulates apoptosis during hepatic I/R injury.

### VHL interacts with HIF-1α and 2α, and HIF-2α plays a protective role in hepatic I/R injury

**FIGURE 4:**
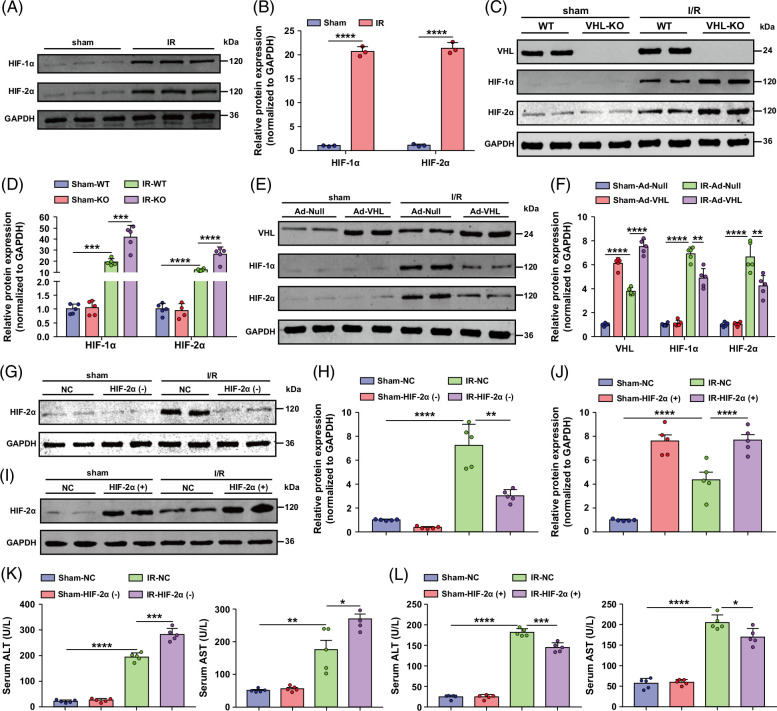
VHL interacts with HIF-1α and HIF-2α in hepatic I/R injury, and the role of HIF-2α in hepatic I/R injury is to reduce liver injury and inflammatory reactions. (A, B) The protein levels of HIF-1α and HIF-2α in the liver after hepatic I/R surgery. (C, D) The protein levels of HIF-1α and HIF-2α in the livers of *VHL*-KO mice after hepatic I/R surgery. (E, F) The protein levels of HIF-1α and HIF-2α in the livers of Ad-VHL mice after hepatic I/R surgery. (G, H) The protein expression level of HIF-2α in the livers of HIF-2α (−) mice and control mice. (I, J) The protein expression level of HIF-2α in the livers of HIF-2α (+) mice and control mice. (K) Serum ALT and AST activities in HIF-2α (−) mice after hepatic I/R surgery. (L) Serum ALT and AST activities in HIF-2α (+) mice after hepatic I/R surgery. GAPDH served as the loading control (n = 5/group). All the data are presented as the means ± SDs. The levels of statistical significance are indicated as follows: *p < 0.05, **p < 0.01, ***p < 0.001, ****p < 0.0001 and ns = not significant. For statistical analysis, 1-way ANOVA with Bonferroni post hoc correction or Tamhane’s T2 post hoc test and 2-tailed Student *t* test were used. Abbreviations: GAPDH, glyceraldehyde-3-phosphate dehydrogenase; HIF, hypoxia-inducible factor; I/R, ischemia and reperfusion; VHL, Von Hippel-Lindau; VHL-KO, VHL gene knockout.

VHL can act on various substrates to exert its functions.^30^^–^^32^ To analyze the role of HIF-1α and 2α in hepatic I/R injury, we first evaluated their protein expression in WT mice subjected to hepatic I/R injury. The levels of HIF-1α and 2α in the liver lobes of WT mice were distinctly increased after hepatic I/R (Figures 4A, B), and the same trend was observed in vitro (Supplemental Figures S1A, B, http://links.lww.com/HC9/B75). We found that HIF-1α and HIF-2α were dramatically increased in the livers of VHL-KO mice compared with those of WT mice after hepatic I/R operation, whereas HIF-1α and 2α were suppressed in the livers of Ad-VHL mice (Figures 4C–F). We used VHL knockout and VHL-overexpressing primary hepatocytes to explore whether VHL regulates HIF-1α and 2α during hepatocyte H/R insult. Similarly, VHL inhibited the expression of HIF-1α and HIF-2α in hepatocytes during H/R insult (Supplemental Figures S1C–F, http://links.lww.com/HC9/B75).

**FIGURE 5:**
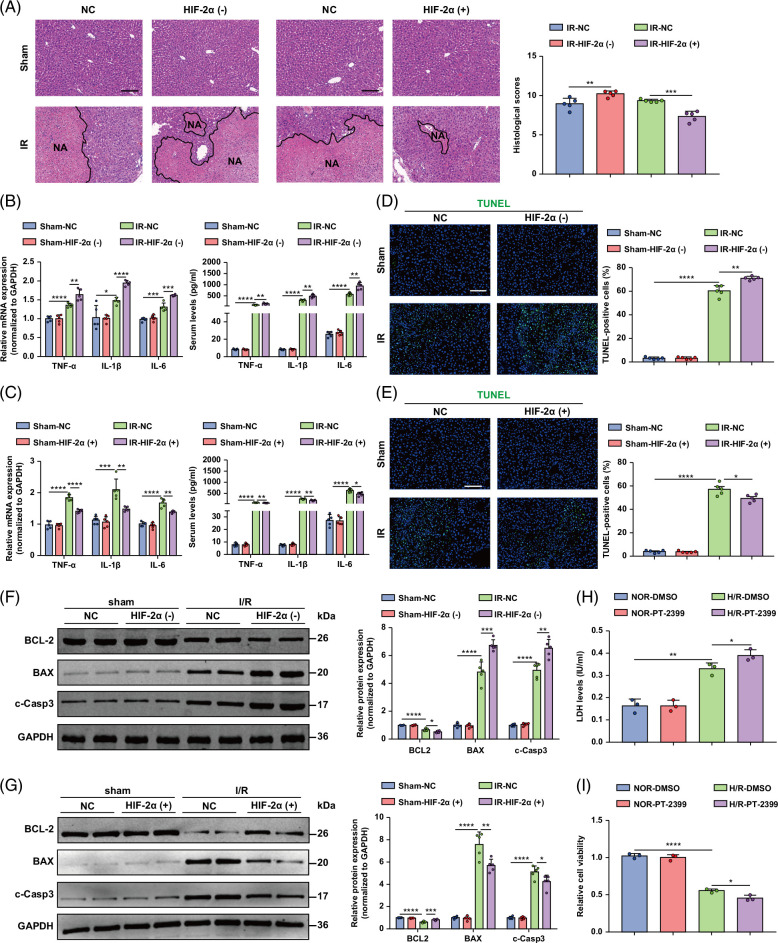
HIF-2α alleviates liver injury and cell death in hepatic I/R injury. (A) Representative histological H&E-stained images showing necrotic areas in liver tissue from HIF-2α (−) and HIF-2α (+) mice after hepatic I/R surgery. Scale bar, 100 μm. (B) The tissue mRNA expression and serum levels of inflammatory cytokine/chemokine genes in the livers of HIF-2α (−) mice and control mice. (C) The mRNA expression and serum levels of inflammatory cytokine/chemokine genes in the livers of HIF-2α (+) mice and control mice. (D) TUNEL staining of liver sections from HIF-2α (−) mice after the I/R operation. Scale bar, 50 μm. (E) TUNEL staining of liver sections from HIF-2α (+) mice after the I/R operation. Scale bar, 50 μm. (F) The protein levels of cell apoptosis–related genes in the livers of HIF-2α (−) mice after I/R surgery. (G) The protein levels of cell apoptosis–related genes in the livers of HIF-2α (+) mice after I/R surgery (n = 5/group). (H) LDH release assay of hepatocytes from PT-2399 hepatocytes after H/R injury. (I) The cell viability index of PT-2399 hepatocytes after H/R injury was determined through a CCK-8 assay. Data are representative of 3 independent experiments. GAPDH served as the loading control. All the data are presented as the means ± SDs. The levels of statistical significance are indicated as follows: *p < 0.05, **p < 0.01, ***p < 0.001, ****p < 0.0001 and ns = not significant. For statistical analysis, 1-way ANOVA with Bonferroni post hoc correction or Tamhane’s T2 post hoc test and 2-tailed Student *t* test were used. Abbreviations: CCK-8, cell counting kit-8; GAPDH, glyceraldehyde-3-phosphate dehydrogenase; H&E, hematoxylin and eosin; HIF, hypoxia-inducible factor; H/R, hypoxia/reoxygenation; I/R, ischemia and reperfusion; LDH, lactate dehydrogenase; TUNEL, TdT-mediated dUTP nick-end labeling.

HIF-2α-knockdown and HIF-2α-overexpressing mice were generated through tail vein injection of adenovirus, and the efficiency of the adenovirus was confirmed by Western blotting (Figures 4G–I). Serum ALT and AST levels were significantly greater in HIF-2α-knockdown mice than in control mice after hepatic I/R. Compared with those in control mice, HIF-2α overexpression significantly decreased the serum ALT and AST levels at 6 hours after liver I/R surgery (Figures 4K, L). Histological analysis showed that HIF-2α knockdown mice exhibited greater necrotic areas after liver I/R surgery than control mice, and we found that HIF-2α-overexpressing mice had a reduced necrotic area after hepatic I/R injury compared with the corresponding controls (Figure 5A). Compared with control hepatocytes, HIF-2α-knockdown mice presented higher serum and tissue mRNA levels of inflammatory cytokines/chemokines, including TNF-α, IL-1β, and IL-6, after liver I/R injury (Figure 5B). In contrast, HIF-2α overexpression resulted in less inflammatory cytokine/chemokine production than that in the control group (Figure 5C).

TUNEL staining revealed that, compared with control mice, HIF-2α-knockdown mice exhibited severe hepatocyte apoptosis after liver I/R insult. However, HIF-2α overexpression attenuated hepatocyte apoptosis (Figures 5D, E). Compared with those in control mice, the expression of the antiapoptotic gene Bcl-2 was significantly reduced, and the levels of the proapoptotic genes Bax and c-caspase-3 were increased in the livers of HIF-2α-knockdown mice during I/R injury (Figure 5F). HIF-2α overexpression had the opposite effect (Figure 5G). In vitro, we detected more LDH release from HIF-2α-knockdown hepatocyte cultures than from hepatocytes. In sharp contrast, HIF-2α-overexpressing hepatocytes exhibited less LDH release than did antithetical hepatocytes (Supplemental Figure S2A, http://links.lww.com/HC9/B75). We further tested the role of HIF-2α in H/R injury by using PT-2399, which can inhibit HIF-2α accumulation. As expected, HIF-2α inhibition by PT-2399 treatment efficiently increased LDH release (Figure 5H). Furthermore, HIF-2α knockdown reduced cell viability, whereas HIF-2α overexpression increased viability in hepatocytes subjected to H/R injury (Supplemental Figure S2B, http://links.lww.com/HC9/B75). PT-2399 treatment also reduced cell viability after H/R injury (Figure 5I). HIF-1α-knockdown and HIF-1α-overexpressing mice were generated through tail vein injection of adenovirus, and the efficiency of the adenovirus was confirmed by Western blotting (Supplemental Figures S3A, B, http://links.lww.com/HC9/B75). We also focused on the biological role of HIF-1α in liver I/R injury in vivo and in vitro. Knockdown of HIF-1α increased liver damage, inflammation, and apoptosis. However, overexpression of HIF-1α had the opposite effect (Supplemental Figures S3C-5B, http://links.lww.com/HC9/B75). These data suggest that VHL can simultaneously regulate HIF-1α and 2α and that HIF-2α plays a protective role in hepatic I/R injury.

### HIF-1α and 2α mediate VHL function in hepatic I/R injury

**FIGURE 6:**
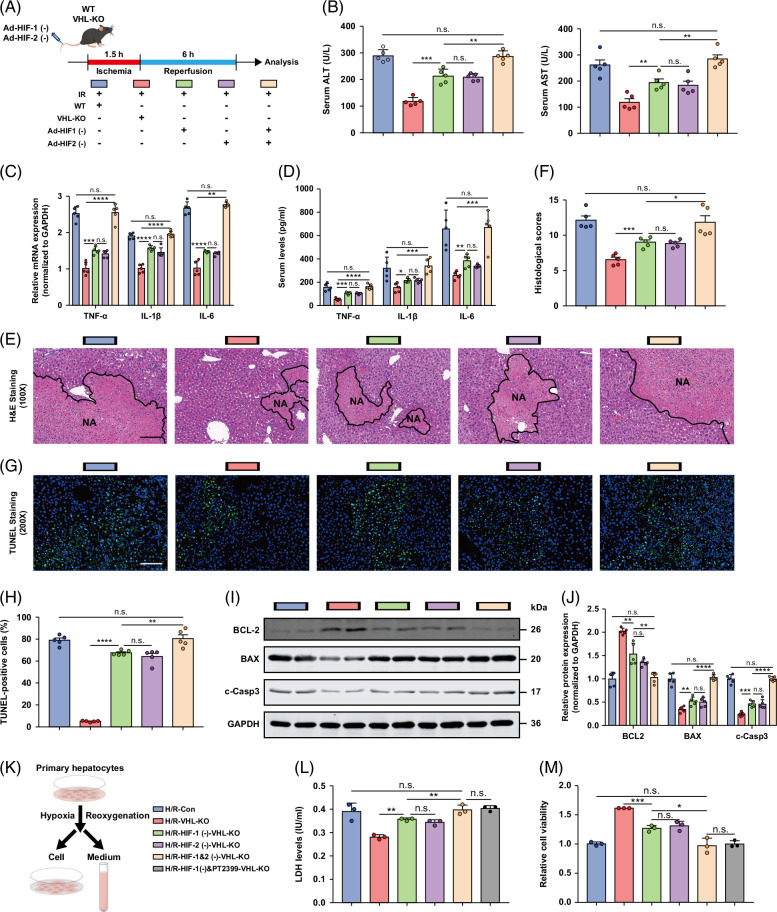
Inhibition of HIF-1α or HIF-2α, as well as concurrent inhibition of HIF-1α and HIF-2α, exacerbates the protective effect of VHL-KO on hepatic I/R injury. (A) Experimental strategy in a mouse model of hepatic I/R injury with inhibition of HIF-1α and 2α accumulation (either individually or simultaneously) in VHL-KO and WT mice. (B) Serum ALT/AST activity in the control group, inhibition of HIF-1α or HIF-2α, and concurrent inhibition of HIF-1α and HIF-2α in VHL-KO mice after hepatic I/R surgery. (C, D) The serum and mRNA levels of inflammatory cytokine/chemokine genes in control, HIF-1α- inhibited or HIF-2α-inhibited, and combined HIF-1α–inhibited and HIF-2α–inhibited VHL-KO hepatocytes challenged with H/R injury (representative of 3 independent experiments). (E, F) Representative histological H&E-stained images showing necrotic areas in liver tissue from the indicated mice after hepatic I/R surgery. Scale bar, 100 μm. (G, H) TUNEL staining of liver sections from control, HIF-1α–inhibited or HIF-2α–inhibited, and combined HIF-1α–inhibited and HIF-2α–inhibited VHL-KO mice after the I/R operation. Scale bar, 50 μm. (I, J) The protein levels of cell apoptosis–related genes in the livers of control, HIF-1α–inhibited or HIF-2α–inhibited, and combined HIF-1α–inhibited and HIF-2α–inhibited VHL-KO mice after I/R surgery (n = 5/group). (K) Experimental strategy in VHL-KO and WT hepatocytes subjected to H/R insult, which inhibited HIF-1α and 2α accumulation (either individually or simultaneously). (L) LDH release assay of VHL-KO hepatocytes with inhibition of HIF-1α or HIF-2α or concurrent inhibition of HIF-1α and HIF-2α (or PT-2399) after H/R injury. (M) The cell viability indices of VHL-KO hepatocytes with inhibition of HIF-1α or HIF-2α or concurrent inhibition of HIF-1α and HIF-2α (or PT-2399) after H/R injury were determined through a CCK-8 assay (representative of 3 independent experiments). GAPDH was used for normalization. All the data are presented as the means ± SDs. The levels of statistical significance are indicated as follows: *p < 0.05, **p < 0.01, ***p < 0.001, ****p < 0.0001 and ns = not significant. For statistical analysis, 1-way ANOVA with Bonferroni post hoc correction or Tamhane’s T2 post hoc test and 2-tailed Student *t* test were used. Abbreviations: CCK-8, cell counting kit-8; GAPDH, glyceraldehyde-3-phosphate dehydrogenase; H&E, hematoxylin and eosin; HIF, hypoxia-inducible factor; H/R, hypoxia/reoxygenation; I/R, ischemia and reperfusion; LDH, lactate dehydrogenase; TUNEL, TdT-mediated dUTP nick-end labeling; VHL-KO, VHL gene knockout; WT, wild-type.

We further tested whether HIF-1α and 2α mediate VHL function in hepatic I/R injury by using 2 types of adenovirus vectors, which inhibit HIF-1α and 2α accumulation, respectively, or simultaneously (Figure 6A). As expected, inhibition of HIF-1α or 2α, as well as concurrent inhibition of HIF-1α and 2α, exacerbated the improvements in ALT and AST levels caused by VHL deficiency (Figure 6B). Inhibition of HIF-1α or 2α, as well as concurrent inhibition of HIF-1α and 2α, potently abrogated the suppressive effect of VHL deficiency on the serum and mRNA expression of proinflammatory cytokines/chemokines (Figures 6C, D). Inhibition of HIF-1α or 2α, as well as concurrent inhibition of HIF-1α and 2α, exacerbated the improvement in liver injury caused by VHL deficiency (Figures 6E, F). Inhibition of HIF-1α or 2α, as well as concurrent inhibition of HIF-1α and 2α, aggravated the decrease in hepatocyte death caused by VHL deficiency (Figures 6G–J). We also explored whether HIF-1α and 2α mediate VHL function in hepatocyte H/R insult (Figure 6K). We found that LDH release was greater in the HIF-1α–inhibited or HIF-2α–inhibited group than in the control group after H/R injury, as well as after concurrent inhibition of HIF-1α and 2α in hepatocyte cultures (Figure 6L). Finally, inhibition of HIF-1α or 2α, as well as concurrent inhibition of HIF-1α and 2α, abrogated VHL-KO function to promote hepatocyte viability (Figure 6M). Interestingly, compared with HIF-1α or 2α knockdown alone, simultaneous knockdown of HIF-1α and 2α significantly reversed the protective effect of VHL-KO on liver I/R injury.

**FIGURE 7:**
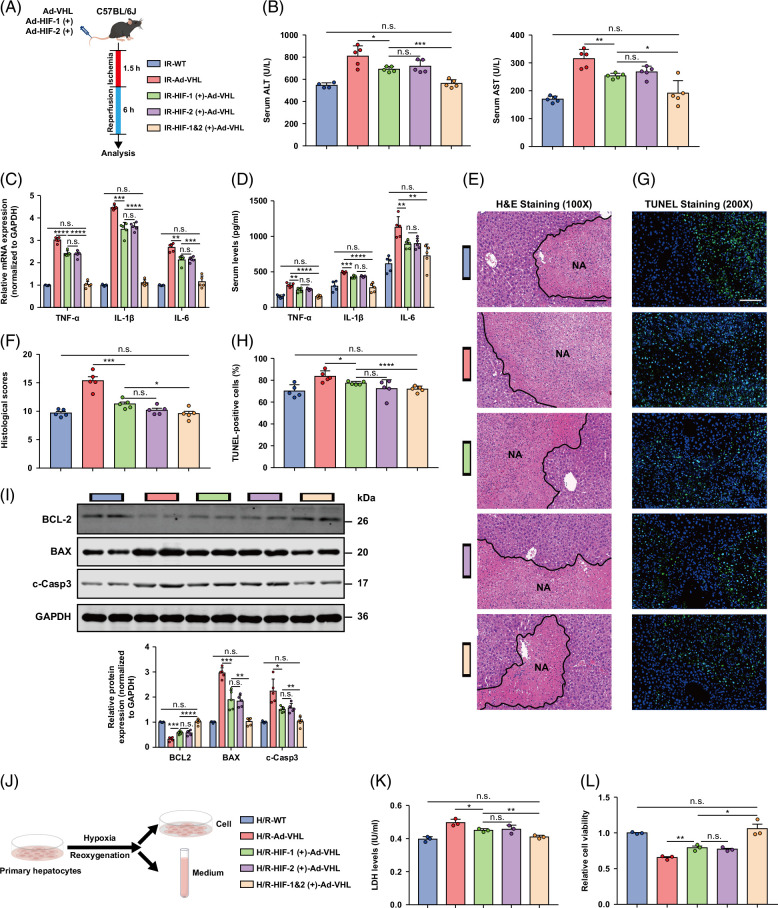
HIF-1α or HIF-2α stabilization and simultaneous overexpression of HIF-1α and HIF-2α compensates for the harmful effect of VHL in hepatic I/R injury. (A) Experimental strategy in a mouse model of hepatic I/R injury in which HIF-1α and 2α are overexpressed (either individually or simultaneously) in Ad-VHL and Ad-Null mice. (B) Serum ALT/AST activities in control mice, Ad-VHL mice overexpressing HIF-1α or HIF-2α, and Ad-VHL mice simultaneously overexpressing HIF-1α and HIF-2α after hepatic I/R surgery. (C, D) The serum and mRNA levels of inflammatory cytokine/chemokine genes in control, HIF-1α–inhibited or HIF-2α–inhibited, and concurrent HIF-1α–inhibited and HIF-2α–inhibited VHL-KO hepatocytes challenged with H/R injury (representative of 3 independent experiments). (E, F) Representative histological H&E-stained images showing necrotic areas in the liver tissues from the indicated mice after hepatic I/R surgery. Scale bar, 100 μm. (G, H) TUNEL staining of liver sections from control, HIF-1α-overexpressing or HIF-2α-overexpressing, and Ad-VHL mice simultaneously overexpressing HIF-1α and HIF-2α after the I/R operation. Scale bar, 50 μm. (I) The protein levels of cell apoptosis–related genes in the livers of control, HIF-1α–inhibited or HIF-2α–inhibited, and concurrent HIF-1α–inhibited and HIF-2α–inhibited VHL-KO mice after I/R surgery (n = 5/group). (J) Experimental strategy for Ad-VHL and Ad-Null hepatocytes under H/R insult in which HIF-1α and 2α were overexpressed (either individually or simultaneously). (K) LDH release assay of hepatocytes from Ad-VHL hepatocytes with overexpression of HIF-1α or HIF-2α or concurrent overexpression of HIF-1α and HIF-2α after H/R injury. (L) The cell viability indices of Ad-VHL hepatocytes with overexpression of HIF-1α or HIF-2α or concurrent overexpression of HIF-1α and HIF-2α after H/R injury were determined through a CCK-8 assay. Data are representative of 3 independent experiments. GAPDH was used for normalization. All the data are presented as the means ± SDs. The levels of statistical significance are indicated as follows: *p < 0.05, **p < 0.01, ***p < 0.001, ****p < 0.0001 and ns = not significant. For statistical analysis, 1-way ANOVA with Bonferroni post hoc correction or Tamhane’s T2 post hoc test and 2-tailed Student *t* test were used. Abbreviations: CCK-8, cell counting kit-8; GAPDH, glyceraldehyde-3-phosphate dehydrogenase; H&E, hematoxylin and eosin; HIF, hypoxia-inducible factor; H/R, hypoxia/reoxygenation; I/R, ischemia and reperfusion; LDH, lactate dehydrogenase; TUNEL, TdT-mediated dUTP nick-end labeling; VHL-KO, VHL gene knockout; WT, wild-type.

In contrast, HIF-1α or 2α stabilization, as well as simultaneous overexpression of HIF-1α and 2α through adenovirus vectors, sufficiently compensated for the harmful effect of VHL on hepatic I/R injury (Figure 7A), as evidenced by decreased ALT and AST levels (Figure 7B), reduced expression of proinflammatory cytokines/chemokines (Figures 7C, D), decreased liver injury (Figures 7E, F), and ameliorated hepatocyte apoptosis (Figures 7G–I). In VHL-overexpressing hepatocytes (Figure 7J), HIF-1α or 2α stabilization, as well as simultaneous overexpression of HIF-1α and 2α reduced LDH release (Figure 7K) and enhanced cell viability (Figure 7L). Our findings indicate that VHL plays a significant role in hepatic I/R injury through HIF-1α/HIF-2α.

**FIGURE 8:**
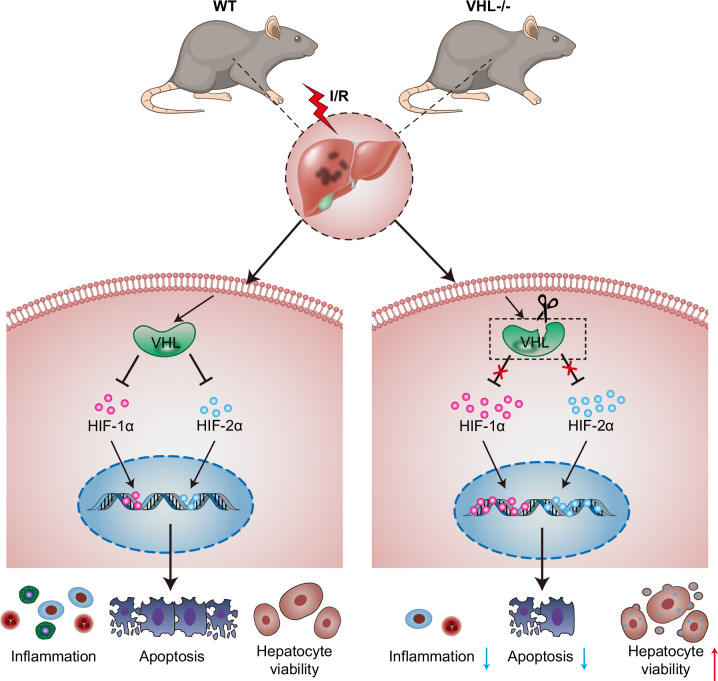
Mechanistic diagram showing that inactivation of VHL protects against hepatic I/R injury through stabilizing HIF-1α and 2α. Abbreviations: HIF, hypoxia-inducible factor; I/R, ischemia and reperfusion.

In brief, all of the above findings illustrated that VHL and HIF-1α/HIF-2α may form a regulatory axis and play important roles in hepatic I/R injury (Figure 8).

## DISCUSSION

In recent years, the pathophysiology and mechanisms of liver I/R injury have been extensively studied and reviewed, generating many potential therapeutic alternatives.^33^ Nevertheless, liver I/R injury remains a serious problem in clinical practice, probably because few basic (or translational) studies have been successful. Therefore, identifying a novel and feasible mechanism for the treatment of hepatic I/R injury is important. Several studies have shown that VHL, an important regulatory factor, is involved in tumors, inflammation, fibrosis, and other pathophysiological processes.^34^^,^^35^ However, the biological and functional roles of VHL in I/R-induced liver damage remain poorly understood. In the present study, we confirmed that VHL upregulation was closely related to hepatic I/R injury in samples from patients with liver resection for benign tumors and in mouse models. Moreover, through genetic methods, we constructed a conditional VHL-KO mouse model based on the tamoxifen-inducible CreERTM system to inactivate the VHL gene and identify the mechanism of hepatic I/R injury. We found that the inhibition of VHL alleviated liver I/R injury, whereas the overexpression of VHL sensitized mice to I/R injury. Taken together, these results suggest that VHL is necessary for the regulation of hepatic I/R injury and that VHL inactivation may be a promising method to protect against I/R-mediated liver damage.

Numerous studies have shown that inflammation is closely involved in hepatic I/R injury.^36^ Recent evidence suggests that apoptosis is another crucial part of liver I/R injury.^37^ Given that sterile inflammation and cell apoptosis are critical markers in the development of hepatic I/R injury, VHL may play important roles in these processes. The inflammatory response during hepatic I/R injury is characterized by the induction of a cascade of proinflammatory mediators that culminates in the recruitment of leukocytes to postischemic tissue, leading to parenchymal cell injury. Consistently, here, we demonstrated that VHL deletion in mice reduced the gene expression of TNF-α, Il-1β, and Il-6 after hepatic I/R injury, whereas VHL overexpression had the opposite effect. Moreover, the same tendency was observed in the in vitro experiments. These results demonstrated that VHL mediates the inflammatory response during liver I/R injury. Apoptosis, also known as programmed cell death, is a physiological cell suicide process that is essential for development and homeostasis. Recent evidence has indicated that apoptosis is another essential step in the pathogenesis of hepatic I/R injury. Our present study demonstrated that VHL deletion ameliorated hepatocyte apoptosis, as indicated by the increased level of the antiapoptotic molecule Bcl-2 and reduced levels of proapoptotic factors such as Bax and c-caspase-3 in the livers of VHL-KO mice after hepatic I/R injury compared with those in WT control mice, whereas VHL overexpression had the opposite effect. Thus, we identified VHL as an efficient regulator of apoptosis in response to hepatic I/R.

The VHL protein (pVHL) mediates an important control mechanism by targeting HIFa subunits for ubiquitin-mediated degradation.^38^ HIFs are composed of an oxygen-dependent α subunit (HIF-1α, 2α, or 3α), a constitutively expressed β subunit, and an aryl hydrocarbon nuclear translocator. Under normoxia, HIF-1α is hydroxylated by proline hydroxylase and then recognized by the VHL protein, resulting in proteasomal degradation.^39^ This process is prevented during hypoxia. HIF-1α can move into the nucleus to form a complex with HIF-1β and CBP/p300 and then induce the transcription of target genes.^40^ HIF-1α is a transcription factor that regulates diverse biological processes, such as tumorigenesis, metabolism, and angiogenesis.^41^ Interestingly, our previous study revealed that HIF-1α is stabilized by VHL.^24^ However, it is not clear whether VHL affects hepatic I/R injury by regulating HIF-1α. In the present study, we verified that HIF-1α plays a protective role in liver I/R injury. We also found that the repression of VHL expression in VHL-KO mice maintained the HIF-1α protein at a considerably high level compared with that in control group mice after I/R operation. In addition, HIF-1α inhibition efficiently repressed the expression of HIF-1α in VHL-KO primary hepatocytes and potently abrogated the protective effect against liver injury in VHL-KO mice. Therefore, the disruption of VHL protects against hepatic I/R injury by regulating HIF-1α.

The HIF-1α subunit was discovered first, followed by the HIF-2α and 3α subunits. While HIF-3α is obviously different from the other 2 subunits, HIF-2α shares 48% amino acid sequence similarity with HIF-1α. Both HIF-1α and 2α activate hypoxia-inducible gene transcription in the same way.^42^ In different cell types, HIF-1α and 2α can play complementary, opposing, or unrelated roles in regulating the response to hypoxic conditions.^43^ Moreover, the role of HIF-2α in the I/R procedure is controversial. For example, Kapitsinou and colleagues demonstrated that HIF-2α is protective against ischemic kidney injury, whereas DeBerge and colleagues demonstrated that HIF-2α aggravated I/R injury in myocardial infarction.^44^^–^^46^ However, the role of HIF-2α and whether it is regulated by VHL during hepatic I/R injury remain unclear. In the present study, VHL overexpression decreased HIF-2α expression during hepatic I/R, whereas VHL knockdown increased HIF-2α expression. Importantly, we first found that HIF-2α plays a protective role in hepatic I/R injury, as evidenced by the finding that the overexpression of HIF-2α reduced liver injury, cell death, inflammatory reactions, and cell viability and that the inhibition of HIF-2α had the opposite effect. HIF-2α inhibition potently abrogated the protective effect of VHL-KO against liver injury, and overexpression of HIF-2α reversed the harmful effects of VHL in hepatic I/R injury. We found that the simultaneous overexpression of both HIF-1α and 2α reversed the detrimental effect of VHL more significantly than the regulation of HIF-1α or 2α alone. Intriguingly, there was no significant difference between HIF-1α and HIF-2α in terms of the effects of VHL in the recovery experiment. Moreover, because we used VHL knockout mice in our study, we cannot exclude the possibility that VHL plays a regulatory role in liver nonparenchymal cells during hepatic I/R injury, which needs further study.

Moreover, the activation of HIF can also be achieved by inhibiting proline hydroxylases, leading to the stabilization of HIF under normoxic conditions. Various classes of compounds that inhibit proline hydroxylase function by preventing cosubstrate 2-OG binding are being investigated. Currently, oral applications of HIF activators such as vadadustat (AKB-6548) and roxadustat (FG-4592) are being tested in clinical settings and have shown promise in reducing liver damage during the perioperative period of liver surgery.^47^^,^^48^ Moreover, the regulation of potential target genes for HIF-1α or 2α could also mediate the observed protection of VHL deletion. For example, under hypoxic conditions, the stabilization of the HIF transcription factors has been demonstrated to govern a repertoire of microRNAs. Recently, miR122 was identified as a target gene of HIF-1α, and pharmacological induction of hepatic miR122 overexpression represents a promising therapeutic strategy for the management of liver I/R injury.^49^^,^^50^

In the majority of cases, extracellular adenosine signaling has anti-inflammatory effects in acute disease states, such as acute lung injury, I/R, and intestinal inflammation. The production of extracellular adenosine increases during inflammatory or hypoxic conditions and is primarily derived from the breakdown of precursor nucleotides, such as ATP. Hypoxia promotes enzymatically controlled ATP/ADP conversion to AMP, which is then converted to AMP by ectonucleoside triphosphate diphosphohydrolase 1 (CD39), followed by conversion to adenosine by ecto-5′-nucleotidase (CD73). Importantly, CD73 is transcriptionally regulated by HIFs. The subsequent activation of adenosine receptors mediates the biological activity of adenosine as a signaling molecule. Adenosine signaling can occur through 4 different G protein–coupled receptors—Adora1, Adora2a, Adora2b, and Adora3—all of which are regulated by HIF-1α or 2α during hypoxia. In summary, HIFs could increase acute adenosine protection during ischemia or inflammation.^51^^,^^52^

In conclusion, HIFs play important roles in liver I/R injury, and our study indicated that the inhibition of VHL is a protective factor against hepatic I/R injury. The protective effects of VHL-KO on hepatic I/R injury are at least partly mediated by the HIF-1α/2α–dependent pathway. Targeting VHL could represent a novel therapeutic approach to significantly dampen hepatic I/R injury and thereby improve outcomes during liver surgery.

## Supplementary Material

**Figure:**